# Next-generation sequencing in advanced Chinese melanoma reveals therapeutic targets and prognostic biomarkers for immunotherapy

**DOI:** 10.1038/s41598-022-13391-y

**Published:** 2022-06-10

**Authors:** Fuxue Huang, Jingjing Li, Xizhi Wen, Baoyan Zhu, Wei Liu, Jiuhong Wang, Hang Jiang, Ya Ding, Dandan Li, Xiaoshi Zhang

**Affiliations:** 1 Biotherapy Center, State Key Laboratory of Oncology in South China, Collaborative Innovation Center for Cancer Medicine, Guangdong Key Laboratory of Nasopharyngeal Carcinoma Diagnosis and Therapy, Sun Yat-Sen University Cancer Center, Guangzhou, 510060 China; 2 State Key Laboratory of Oncology in South China, Collaborative Innovation Center for Cancer Medicine, Guangdong Key Laboratory of Nasopharyngeal Carcinoma Diagnosis and Therapy, Sun Yat-Sen University Cancer Center, Guangzhou, 510060 China; 3 Department of Oncology, The First Affiliated Hospital of Anhui Medical University, Hefei, 230022 China

**Keywords:** Cancer, Skin cancer, Melanoma

## Abstract

Limited studies have interrogated the genomic landscape of Chinese melanoma in which acral and mucosal melanoma are the mainstay. In this study, we carried out a retrospective analysis on 81 Chinese melanoma patients (15 acral, 25 mucosal and 41 cutaneous melanoma). With the identification of 1114 mutations spanning 248 genes, we summarized that the mutation spectrum varied significantly by subtypes. Acral melanoma and mucosal melanoma had significantly more CNVs. *MYC* amplification was one of the most commonly detected CNVs, other frequent CNVs in mucosal melanoma included *NBN* and *KDR*, which were associated with the poor survival of melanoma patients. A generally low TMB, with a median of only 5.1 mut/Mb, was observed in three groups including cutaneous melanoma. Additionally, over 50% variants in DNA damage repair pathway were detected in all three subtypes, most of which were HRD related genes. Patients with alterations of HRD related genes had a longer survival time after immunotherapy. This study revealed a molecular profiling of Chinese patients with advanced melanoma, and proposed the high variant rate in DDR pathway as a biomarker of immunotherapy, which might provide therapeutic targets and guidance in making clinical decision for different Chinese melanoma.

## Introduction

The Cancer Genome Atlas (TCGA) research defined molecular subtypes of cutaneous melanoma on the basis of the presence of specific “driver” gene (*BRAF*, *RAS*, and *NF1*) mutations^1^. In ultraviolet-shielded melanoma, mutations of *BRAF*, *NRAS* or *NF1* are less frequent compared to cutaneous melanoma, but the existence of other cancer driver gene mutations are detected. Accurate profiling of the spectra of mutational changes in melanoma facilitates personalized management of the disease. Due to the subtype bias, the overall mutation frequencies of *BRAF* and *KIT* in Asian patients is approximately 16–25% and 6–10%^2–4^, respectively. However, the overall frequencies of *BRAF* and *KIT* mutations in Caucasians are about 10–60% and 0–28%, respectively^5,6^. More than 50% of Asian patients fail to gain benefits from *BRAF* and *c-KIT* targeted therapy because of the low frequency rates of BRAF and KIT mutations.

Several inhibitors targeting at poly (ADP-ribose) polymerase (PARP) have already been approved by FDA or undergoing clinical trials in various diseases and treatment settings. It has been reported that tumors displaying DNA repair dysfunction might exhibit a *BRCA-like* behavior, according to the concept of “*BRCAness*”^7,8^. It is noteworthy that the efficacy of PARP inhibitors has also been reported in *non-BRCA* related tumors. Tumors with “*BRCAness*” might therefore benefit from PARPi treatment^9–12^. DDR (DNA damage repair) gene alterations ubiquitously exist in many cancer types, and significant enrichment of somatic mutations in DDR genes is approximately detected in 1/3 of TCGA PanCanAtlas cancer types^13^. However, the frequency of DDR gene mutations has been reported to be rare in melanoma, of which most were genes related to UV-induced DNA Damage.

Elucidating gene alteration signatures in all subtypes is crucial for strategic decision in melanoma management. However, the majority of melanoma genomic sequencing data regarding cutaneous melanoma and limited small-cohort studies focused on acral and mucosal melanoma were mainly reported in western countries. In addition, the whole exome sequencing (WES) and whole genome sequencing (WGS) reported in the previous studies are hardly feasible in wide clinical application in China. Therefore, in this study, we conducted a genetic comparison among three subtypes of melanoma with a selected genes panel of next-generation sequencing (NGS) and evaluated its feasibility in practical application.

## Results

### Clinicopathological features

In cutaneous melanoma, 36.6% of patients had congenital nevus and 29.3% of patients had exposure history to sun, which were both significantly higher than patients with acral (p = 0.001) and mucosal melanoma(p = 0.001). While in acral melanoma, 66.7% and 40% of patients had ulceration and trauma respectively, which were higher than that in mucosal and CSD/NCSD (chronic sun-induced damage /non-chronic sun-induced damage) group (p = 0.020 and p = 0.010). Data were shown in Table 1.

**Table 1:** Clinicopathological characteristics.

	CSD/NCSD n = 41 (%)	Mucosal n = 25 (%)	Acral n = 15 (%)	*p* value
Median age (range)	44 (36–59)	53 (48–64)	55 (50–60)	0.085
**Gender (%)**				0.315
Male	23 (56.1)	12 (48.0)	5 (33.3)	
Female	18 (43.9)	13 (52.0)	10 (66.7)	
**Stage (%)**				**0.047**
IA	1 (2.9)	0 (0)	0 (0)	
IIA	1 (2.9)	2 (10)	2 (13.3)	
IIB	5 (14.7)	3 (15.0)	0 (0)	
IIC	3 (8.8)	1(5.0)	5 (33.3)	
IIIA	0 (0)	3(15.0)	0 (0)	
IIIB	2 (5.9)	2(10.0)	0 (0)	
IIIC	15 (44.1)	3(15.0)	5 (33.3)	
IV	7 (20.6)	6 (30.0)	3 (20.0)	
**Sample type (%)**				0.584
Primary	35(85.4)	21 (84.0)	11 (73.3)	
Metastatic	6(14.6)	4 (16.0)	4 (26.7)	
**Ulceration (%)**				**0.020**
0	30 (73.2)	17 (68.0)	5 (33.3)	
1	11 (26.8)	8 (32.0)	10 (66.7)	
**Congenital nevus (%)**				**0.001**
0	26 (63.4)	25 (100.0)	14 (93.3)	
1	15 (36.6)	0 (0)	1 (6.7)	
**Trauma (%)**				**0.014**
0	34 (82.9)	24 (96.0)	9 (60.0)	
1	7 (17.1)	1 (4.0)	6 (40.0)	
**Sun exposure (%)**				**0.001**
0	29 (70.7)	25 (100.0)	15 (100.0)	
1	12 (29.3)	0 (0)	0 (0)	

*P values < 0.05 in bold are statistically significant.

### Alterations of driver genes of cutaneous, mucosal and acral melanoma

**Figure 1:**
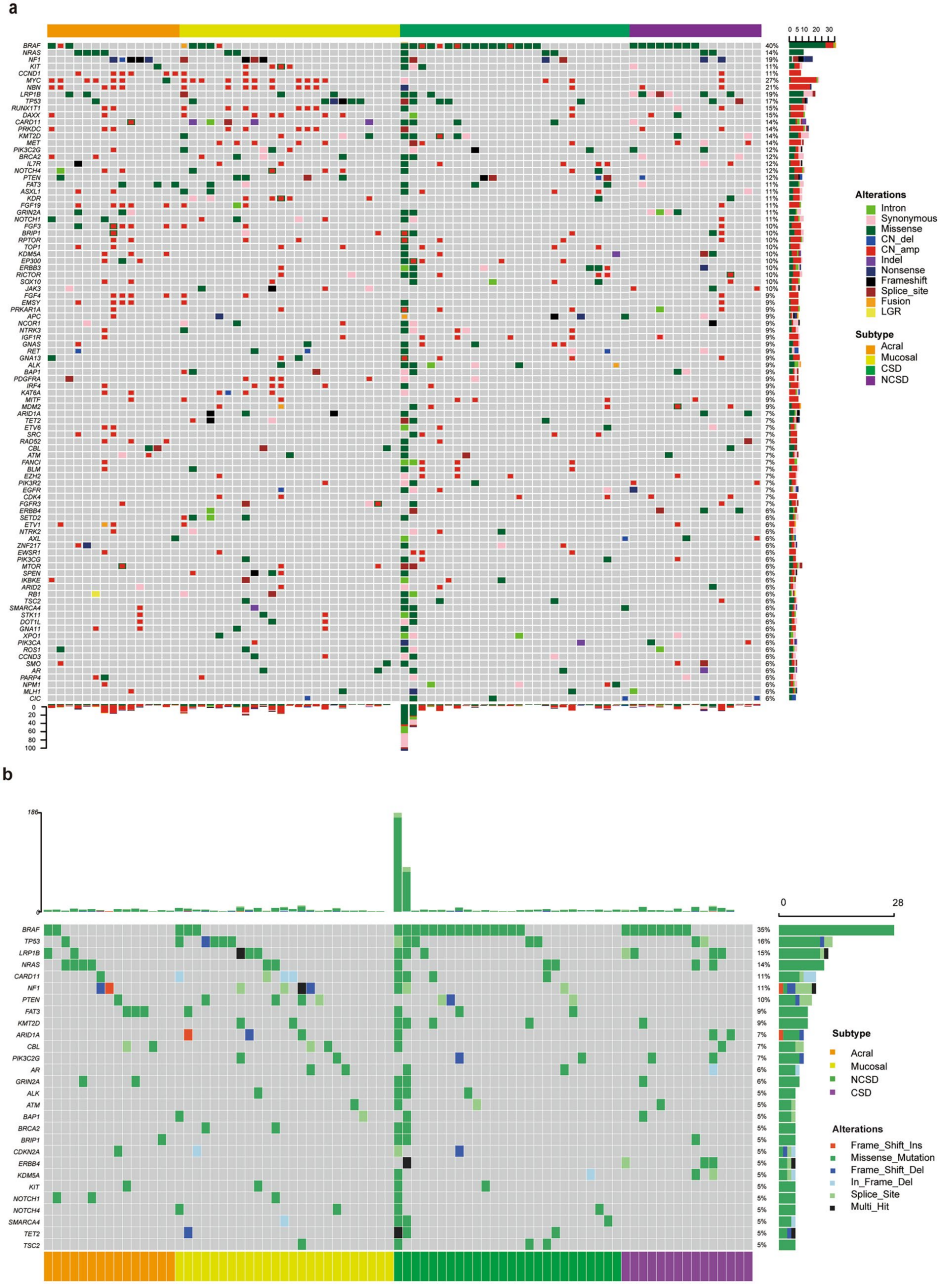
Genomic profiles and mutation characteristics of cutaneous, mucosal and acral melanoma. Each column represents an individual tumor underwent NGS. (**a**) Genomic profiles of 3 subtypes of melanoma (cutaneous melanoma means CSD and NCSD), including 95 genes with variant frequency over 5%. Alterations colored by different types. Main driver genes (BRAF, NRAS, NF1, KIT) were shown on the top of heatmap. (**b**) Oncoplot of mutations in mutated genes with mutation rate over 5%.

A total of 1114 mutations spanning 248 genes were found in 81 Chinese melanoma patients, in which 95 genes were detected with frequency over 5% (Fig. 1a). Mutations of 28 genes were observed in at least 5% patients (Fig. 1b). In cutaneous (CSD/NCSD) melanoma, the most frequently mutated gene was *BRAF* (56.1%, 23/41), which was similar to that in western population. Among patients with *BRAF* mutations, *BRAF-V600E/K* mutation was found in 22 patients, p.P402L mutation was detected in one patient, and *BRAF* amplification was observed in 4 patients. The 7 cases of *NRAS* mutations were targeted to hotspots on codon 61 and codon 12, which were hot spot mutations of cutaneous melanoma. *KIT* mutations on p.Y936 and p.A89T were found in 2 cases. *NF1* mutations were observed in 3 patients (7.3%), in which 1 was missense mutation and 2 were splicing variants. Driver gene mutations were not found in 17 (41.5%,17/41) patients. *LRP1B* (17.1%, 7/45) was the most frequently mutated gene after *BRAF* in cutaneous melanoma, which was equal to *TP53* (17.1%,7/45).

In mucosal melanoma, the mutation frequency of *BRAF* was 12.0% (3/25), which was significantly lower than that in cutaneous melanoma (*p* = 0.001). In addition, a case of *BRAF* amplification and a case of *KCTD15-BRAF* fusion were observed. Two cases (8.0%) had mutations in *NRAS* of p.Q61R and p.G12D, which were consistent with hot spots in cutaneous melanoma. All 4 cases of *KIT* amplifications were found in mucosal melanoma, among which 1 case was co-occurred with p.W557G mutation. *NF1* mutations were observed in 4 patients (16.0%), including missense mutations in 2 patients and splicing variants in 2 patients. *TP53* (20.0%, 5/25) was the gene with the highest mutation frequency in mucosal melanoma.

In acral melanoma, *BRAF* mutation was observed in 2 (13.3%, 2/15) patients, which was significantly lower than that in cutaneous melanoma (*p* = 0.006). Both of them were *BRAF-V600* mutations. *BRAF* amplification was observed in 1 patient. *NRAS* (26.7%, 4/15) was the gene with the highest mutation frequency in acral melanoma, including 2 p.Q61K and 2 p.Q61R mutation. One patient (6.7%) was observed with *KIT* mutation at p.K642E, and 2 patients (13.3%) had *NF1* splicing variants. Driver gene mutations were not found in 7 (46.7%, 7/15) patients. *FAT3* mutation was observed in 20% (3/15) patients with acral melanoma, ranking only second to *NRAS*.

### Somatic copy number alterations were more common in acral and mucosal melanoma and predicted worse survival

**Figure 2:**
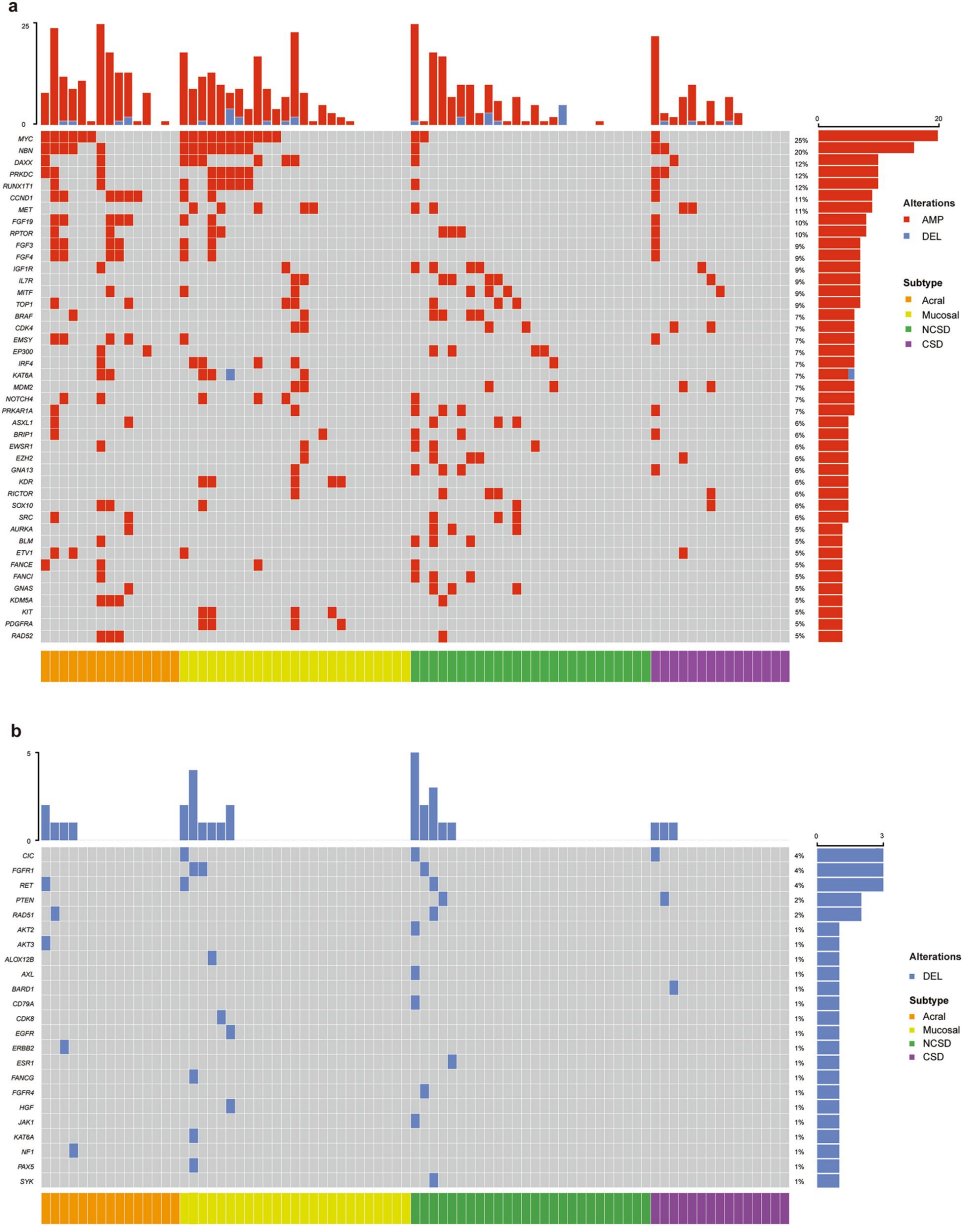
Somatic copy number alterations (CNVs) of cutaneous, mucosal and acral melanoma. The changes in copy number of 3 subtypes. (**a**,**b**) The Gain (CN > 2.25 was regarded as hotspot genes, and CN > 2.5 was counted as others) was represented in red, while the Loss (CN < 1.75 was regarded as hotspot genes, CN < 1.5 was counted as others) was displayed in blue.

**Figure 3:**
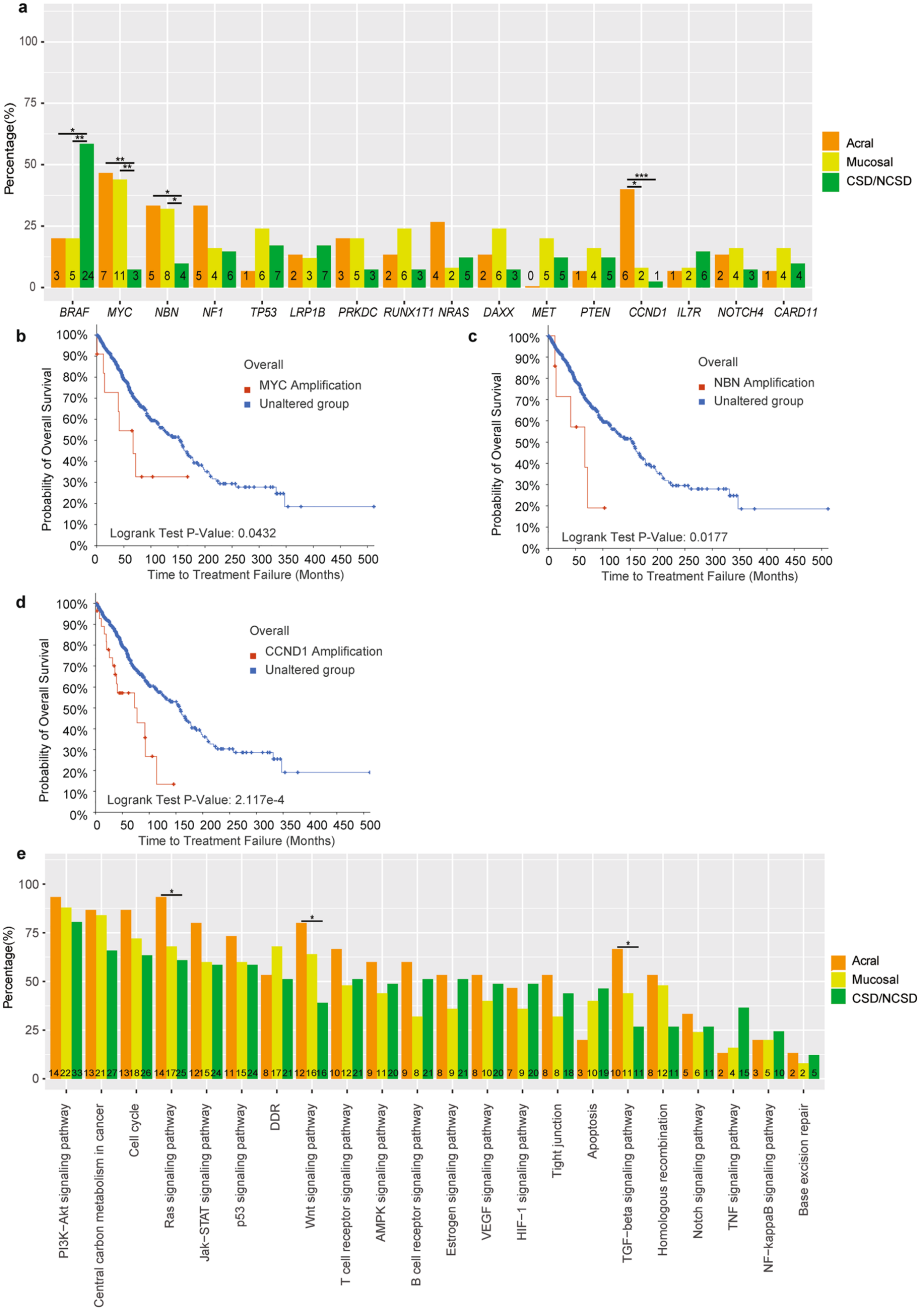
Comparison of variant genes and variant pathways in cutaneous, mucosal and acral melanoma. (**a**) Comparison of variant genes in 3 subtypes (variants that observed over 8 people were showed). (**b**–**d**) Association of *MYC*, *KDR*, and *CCND1* amplification with melanoma patient survival in cBioPortal dataset (MSKCC, Clin Cancer Res 2021, n = 696)^14^. (**e**) Comparison of pathway variants, out of which BRAF was excluded due to its highest mutation frequency. Pathway comparison was based on the KEGG Pathway database. Fisher’s exact test was performed, and the statistical significance was defined as *p < 0.05, **p < 0.01, ***p < 0.001.

Genes were then identified with copy number variations (CNVs). Mucosal and acral melanoma had significantly more CNVs (48.3% and 63.2%) than cutaneous melanoma (19.7%). Genes with CNVs in three groups were shown in Fig. 2a,b. *MYC* amplification was verified to most commonly occur in mucosal and acral melanoma, with frequencies of 44.0% (11/25) and 40.0% (6/15) respectively, which were significantly higher than that in cutaneous melanoma (3/41, 7.3%, *p* = 0.001 and *p* = 0.008). *NBN* followed *MYC* had the highest amplification rate in mucosal group (8/25, 32.0%) and acral group (5/15, 33.3%), compared to that in cutaneous melanoma (4/41, 9.8%, *p* = 0.045 and *p* = 0.048) (Fig. 3a). We then analyzed the survival data of melanoma patients from cBioPortal database and found that patients with *MYC* and *NBN* amplification had a significantly shorter survival time **(**MSKCC, Clin Cancer Res 2021, n = 696, Fig. 3b,c)^14^. *CCND1* amplification was found in 40.0% (6/15) patients in acral group, which was higher than that in mucosal group (8%, 2/25, *p* = 0.036) and cutaneous group (2.4%, 1/41, *p* = 0.001) (Fig. 3a). *CCND1* amplification also predicted a poor survival of melanoma patients (MSKCC, Clin Cancer Res 2021, n = 696, Fig. 3d)^14^. 77.8% *CCND1* amplification co-occurred with *FGF3/4/19* amplification (11q13.3). *KDR* and *KIT* amplifications both occurred in mucosal group. While deletions were less found in our cohort, and none of their frequencies was over 5%. The most commonly deleted genes were *CIC*, *FGFR1*, and *RET*, with equal frequency of 3.7% (Fig. 2b).

### Comparison of variants in pathways

According to the classification of KEGG Pathway database, we compared the gene alterations in different pathways among three groups. Since *BRAF* was found to possess the highest mutation frequency, MAPK pathway was consequentially considered as the most activated pathway, thus *BRAF* was removed from the comparison of each pathway. All of the three groups had more than 75.0% PI3K-AKT pathway mutations. Acral group showed 93.3% (14/15) mutations in Ras pathway, which was higher than that in cutaneous melanoma (61.0%, 25/41, *p* = 0.023). Acral melanoma also had more mutations in WNT pathway and TGF-beta pathway (80.0%, 12/15 and 66.7%, 10/15), compared with that in cutaneous melanoma patients (39.0%, 16/41, *p* = 0.014 and 26.8%, 11/41, *p* = 0.01) (Fig. 3e).

Meantime, all of the three subtypes had more than 50% variants in DNA damage repair pathway. Mutations in DDR related genes were mainly *BRCA1* (1/81), *BRCA2* (4/81), *ATM* (4/81), *PALB2* (2/81), *CHEK2* (2/81), *BAP1* (4/81) and *IDH1* (1/81), which were reported to possibly response to PARP inhibitors.

### Association of TMB and gene alterations with prognosis of immunotherapy

Among 81 patients enrolled, 25 of them received palliative immunotherapy including anti-PD-1 antibody (pembrolizumab or nivolumab), anti-CTLA4 antibody (ipilimumab) monotherapy or combinations. The clinicopathological information of patients receiving first-line immunotherapy was shown in Table S2.

**Figure 4:**
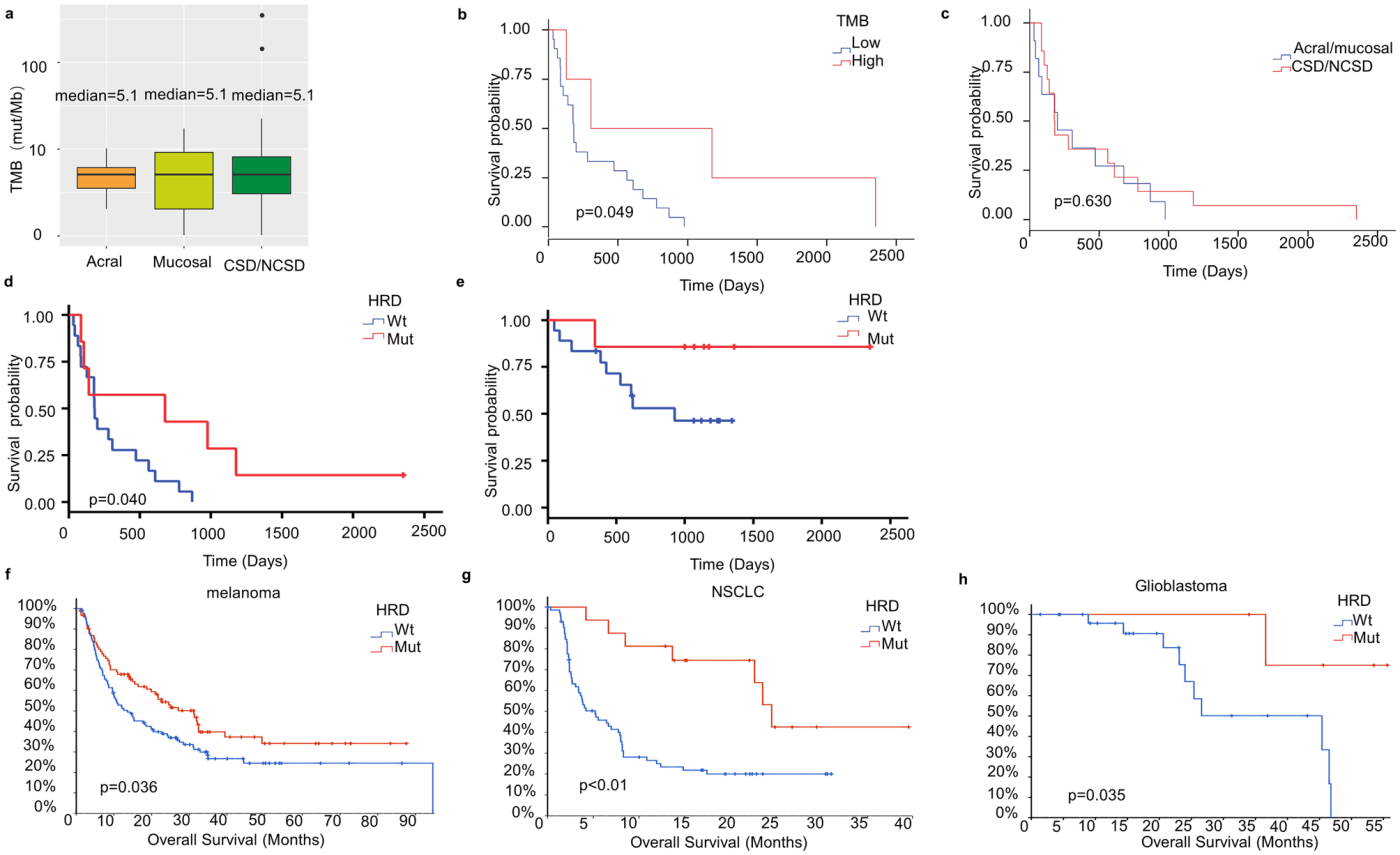
Comparison of TMB in three subtypes and genes related to immunotherapy effect. (**a**), TMB in cutaneous, mucosal and acral melanoma (p = 0.692, Mann–Whitney test). (**b**–**g**), Kaplan Meier assessments of progression-free survival (PFS) in patients treated with immunotherapy in our cohort (n = 25) according to the following classes: b, high TMB (> 15 mut/Mb) and low TMB (≤ 15 mut/Mb); (**c**) cutaneous group with acral/mucosal group; (**d**) PFS of HRD wt and HRD mut; (**e**) OS of HRD wt and HRD mut (HRD related genes: *CHEK1, CHEK2, BRCA1, BRCA2, ATM, PALB2, BAP1, IDH1*); f–h, Association of HRD mut with patient survival of melanoma (UCLA, Cell 2016 + MSKCC, NEJM 2014 + DFCI, Science 2015, n = 202)^15–17^, NSCLC (MSK, Cancer Cell 2018, n = 75)^18^ and glioblastoma (Columbia, Nat Med. 2019, n = 42)^19^ after immunotherapy in cBioPortal dataset.

The median of TMB in three groups was 5.1mut/Mb (Fig. 4a). We derived a cutoff of 15mut/Mb, which can effectively distinguish clinical response. Patients with TMB > 15 mut/Mb had a significantly longer PFS than patients with TMB ≤ 15 mut/Mb (*p* = 0.049) (Fig. 4b). High TMB was associated with improved PFS.

Then mucosal and acral melanoma which are dominant in Chinese population were combined into one group for further comparison, namely Acral/Mucosal group and CSD/NCSD group. We found that PFS in CSD/NCSD group was similar to that in acral/mucosal group (*p* = 0.711), as shown in Fig. 4c. Because of the high alteration rate of DDR pathway in our cohort, especially in HRD related genes (*CHEK1, CHEK2, BRCA1, BRCA2, ATM, PALB2, BAP1, IDH1*), patients were divided into two groups, and we found that patients with alterations of HRD related genes showed a longer PFS (9.7 *vs.* 25.3 months, *p* = 0.040) and a prolonged survival time after immunotherapy (*p* = 0.117) (Fig. 4d,e). Then we validated our findings by other researches. This finding was then validated by gene mutation data of patients in other melanoma groups (UCLA, Cell 2016 + MSKCC, NEJM 2014 + DFCI, Science 2015, n = 202)^15–17^ and patients with NSCLC (MSK, Cancer Cell 2018, n = 75)^18^ and glioblastoma (Columbia, Nat Med. 2019, n = 42)^19^ who accepted immunotherapy, taken from a publicly available database (CBioPortal) (Fig. 4f–h). DDR pathway alterations may serve as a biomarker of immunotherapy.

## Discussion

In the current study, we performed NGS sequencing with a multiple-gene panel to investigate the comprehensive molecular characterization of 81 Chinese melanoma patients and evaluated the clinical correlations of gene status, we also evaluated the correlations between the response of immunotherapy and gene alterations. This study aimed to exploit tumor targeted NGS to compare different melanoma types in Chinese population. The mutation landscape of Chinese melanomas differed from that of western population, which was distinguished by melanoma types. It may provide signposts for the identification of drugable targets and predictive biomarkers, as well as potentially useful guidance for proper therapeutic decisions regarding different types of Chinese melanoma.

Cutaneous melanoma in western population is classified into 4 molecular subtypes based on the pattern of the most significantly mutated genes, namely mutant *BRAF* (52%, 166/318), mutant *RAS* (28%, 88/318), mutant *NF1* (14%, 46/318), and Triple-WT (wild-type) (14%, 46/318)^1^. In our study, mutant *BRAF* (51.1%,23/45) of cutaneous melanoma in Chinese was similar to that in Caucasians, and *BRAF V600E/K* was the commonest mutation. Similar to other studies in Asian patients, we had a lower mutant *RAS* (15.6%, 7/45) and mutant *NF1* (7.3%, 3/45)^20^, while Triple-WT was higher (37.8%, 17/45). Mucosal and acral melanoma are the main subtypes of Chinese melanoma. Compared with cutaneous melanoma, *BRAF* mutations were less observed in acral melanoma, while mutant *NRAS* (26.7%) and Triple-WT (7/15, 46.7%) were more common. In mucosal melanoma, mutations in *BRAF* (12.0%) and *NRAS* (8.0%) were both lower than that in western population, and lower than Chinese cutaneous melanoma. *NF1* mutation (16.0%) of mucosal melanoma was similar to that in western population, but higher than that of Chinese cutaneous melanoma. Consistent with previous studies, our study further confirmed that gene mutations were more common in cutaneous melanoma, while more CNVs were observed in mucosal and acral melanoma^1,21,22^. In our study, the most common CNVs in mucosal and acral melanoma was *MYC* amplification, consistent with previous study in Asian patients^20^. In vitro experiments have confirmed that high expression of *c-MYC* is positively correlated with the aggressiveness of cutaneous melanoma, and the inhibitor can effectively inhibit tumor growth^23^. Although the role of *MYC* in the development and progression of mucosal and acral melanoma remains to be further studied, *MYC* may act as a new therapeutic target for the treatment of mucosal and acral melanoma with the application of effective inhibitors. *NBN* followed *MYC* also had a high CNVs rate in mucosal and acral melanoma. *NBN* is an important gene which is related to DNA damage repair, and plays an important role in protecting chromosome integrity. Currently, the variation and role of *NBN* in melanoma has not been reported. The variation frequency of *KDR* (*VEGFR-2*) in mucosal melanoma was only second to that of *MYC* and *NBN*. *KDR* is a main functional receptor of VEGF which plays a role in angiogenesis growth of tumor cells^24^. The cBioPortal database showed that *MYC*, *NBN* and *KDR* predicted poor survival of melanoma patients. Guo Jun et al. reported that Axitinib combined with PD-1 antibody achieved ORR at 48.3% in mucosal melanoma^25^, suggesting that Axitinib and other multi-target small molecule inhibitors targeting at angiogenesis may be used in mucosal melanoma and as a combination to improve the efficacy of immunotherapy. Guo Jun et al. reported that genetic aberrations in the cyclin-dependent kinase (CDK)4 pathway occur in 82% of patients with acral melanoma, showed *CDK4* gain (39.5%) and *CCND1* gain (26.7%). *CCND1* amplification was found in 40.0% (6/15) patients in our acral group, similar to their report. Previous studies have reported that, *TERT* promoter mutations are more common in acral and mucosal melanoma patients^21^, and *SF3B1* mutation are more common in mucosal melanoma, mainly in Caucasians^21^. However, because of the limitation of our panel, these genes are not detected in our study.

Previous studies have shown that patients with higher TMB were more likely to gain a better efficacy of immunotherapy^16,26–28^. Pembrolizumab has been approved by the FDA for the treatment of solid tumors with TMB over 10 mut/Mb^29^. TMB plays an increasingly important role in immunotherapy. However, in our study, a generally low TMB was observed in Chinese melanoma patients, and the median of TMB was only about 5.1 mut/Mb, with no difference found in cutaneous, mucosal and acral melanoma. In KEYNOTE-151, the overall response rate of second-line Pembrolizumab for Chinese patients was 16.7%, and first line anti-PD-1 antibody for Chinese patients was 15.6%, both of which were lower than that of western population^30–32^. The generally low TMB in Chinese population and poor immunogenicity might be the reason why the efficacy of immunotherapy in Chinese population, including cutaneous melanoma, is worse than that in western population.

PARP inhibitors are considered to be effective drugs for the treatment of *BRCA* germline mutations. It has been reported that PARP inhibitors also play a role in tumors with no *BRCA* germline mutations but *BRCA* somatic mutations or other HRD mutations^9,10,13^. In our study, we observed a high mutation frequency at 14.8% in HRD pathway, except for *BRCA1* (1/81), *BRCA2* (4/81), other genes like *ATM* (4/81), *PALB2* (2/81), *CHEK2* (2/81), *BAP1* (4/81) and *IDH1* (1/81) were included. It is reported that patients with homologous recombination deficiency (HRD) pathway mutations usually had an increased burden of neoantigens^33^. PARP inhibitors may be applied to patients with these variants to further improve the efficacy of immune checkpoint inhibitors.

To conclude, we compared the mutation profiling of three main subtypes of Chinese melanoma. We observed a generally low TMB of Chinese melanoma patients, but a high variants rate in DDR pathway, especially in HRD related genes, which may contribute to the exploration of new drugable targets. Additionally, this study highlighted the importance of implementing next-generation sequencing testing.

## Methods

### Ethics statement

This study was conducted in accordance with the 1964 Helsinki Declaration. All human studies were approved by the Ethics Committee of Sun Yat-sen University Cancer Center (GZR2017-207). Written informed consent was obtained from eligible patients.

### Patient selection and sample collection

Formalin-fixed paraffin-embedded tissues were obtained from 84 Chinese patients diagnosed with melanoma between September 2017 and September 2021 in the authors’ clinic in Sun Yat-sen University Cancer Center. Standard histopathology was performed to confirm the diagnosis of malignancy and histologic subtype. Among 84 analyzed cases, 3 samples were excluded due to insufficient DNA quantity. A total of 81 melanoma cases were enrolled, including 25 (30.9%) mucosal melanoma, 15 (18.5%) acral melanoma and 41 (50.6%) cutaneous melanoma (namely CSD and NCSD melanoma). The median age of patients of each subtype at diagnosis was 44, 53 and 55 years old respectively.

### Tissue DNA extraction, NGS detection and sequencing data analysis

DNA was extracted with the QIAamp DNA FFPE tissue Kit (Qiagen) according to the manufacturer's instructions. DNA concentration was measured by Qubit dsDNA assay.

Genetic profiles of all tissue samples were assessed by performing capture-based targeted deep sequencing with the OncoScreen panel (Burning Rock Biotech Ltd.) which covered 2.02 MB of human genomic regions, including all exons and critical introns of 295 genes, and genes included in the panel were listed in Table S1. Details of sequencing data analysis were described as previously reported^34^. The calculation of tumor mutation burden (TMB) was based on the ratio of the total number of mutations to the size of panel.

### Validation data collection and analysis

The data of targeted gene mutations and survival data were obtained from cBioPortal database, and analyzed on cBioPortal, samples were divided into ‘mut’ or ‘wt’. Gene mutation data and survival data of *MYC*, *NBN* and *CCND1* were from the study of Melanoma (MSKCC, Clin Cancer Res 2021, n = 696)^14^. DDR related gene (*CHEK1, CHEK2, BRCA1, BRCA2, ATM, PALB2, BAP1, IDH1*) mutation data and survival data of patients treated by immunotherapy were also obtained from cBioPortal, melanoma (UCLA, Cell 2016 + MSKCC, NEJM 2014 + DFCI, Science 2015, n = 202)^15–17^, NSCLC (MSK, Cancer Cell 2018, n = 75)^18^ and glioblastoma (Columbia, Nat Med. 2019, n = 42)^19^. P < 0.05 was considered to indicate a statistically significant difference.

### Statistical analysis

Patient follow-up data were acquired from medical records. The χ^2^ test and Fisher’s exact tests were applied to analyze the association. The significance of the association of the mutations between the three groups was analyzed using Fisher’s exact test. The Mann–Whitney test was used to compare tumor mutation burden. The Kaplan–Meier method was utilized to conduct survival analysis. All statistical analyses were accomplished by SPSS V.20.0 software. P value < 0.05 was considered to be statistically significant.

## Supplementary Information

Supplementary Table S1.

Supplementary Table S2.

Supplementary Information 3.

Supplementary Information 4.

Supplementary Information 5.

## Data Availability

The datasets generated and/or analyzed during the current study are available in the Figshare repository, https://doi.org/10.6084/m9.figshare.19115486.
