# Supplementary material for: Next-generation sequencing in advanced Chinese melanoma reveals therapeutic targets and prognostic biomarkers for immunotherapy
Source: Sci Rep. 2022 Jun 10;12:9559. doi: 10.1038/s41598-022-13391-y (PMC9187737; doi:10.1038/s41598-022-13391-y)
Supplement: Supplementary file 1 — Supplementary Table S1. [file 41598_2022_13391_MOESM1_ESM.docx]

**Next-generation Sequencing in advanced Chinese melanoma reveals therapeutic targets and prognostic biomarkers for immunotherapy**

Fuxue Huang^#1,2^, Jingjing Li^#1^, Xizhi Wen^#1^, Baoyan Zhu^1,3^, Wei Liu^1^, Jiuhong Wang^1^, Hang Jiang^1^, Ya Ding^*1^, Dandan Li^*1^, Xiaoshi Zhang^*1^

| 295 genes of the NGS panel | | |  |  |  |  |  |  |  |
| --- | --- | --- | --- | --- | --- | --- | --- | --- | --- |
| *ABL1* | *BRAF* | *CHEK2* | *ETV4* | *FGFR4* | *JAK2* | *MRE11A* | *PALB2* | *RAD51D* | *STAG2* |
| *AKT1* | *BRCA1* | *CHUK* | *ETV5* | *FLT1* | *JAK3* | *MSH2* | *PARP1* | *RAD52* | *STAT4* |
| *AKT2* | *BRCA2* | *CIC* | *ETV6* | *FLT3* | *JUN* | *MSH6* | *PARP2* | *RAD54L* | *STK11* |
| *AKT3* | *BRIP1* | *CRBN* | *EWSR1* | *FLT4* | *KDM5A* | *MTOR* | *PARP3* | *RAF1* | *SUFU* |
| *ALK* | *BTG1* | *CREBBP* | *EZH2* | *FOXL2* | *KDM5C* | *MUTYH* | *PARP4* | *RARA* | *SYK* |
| *ALOX12B* | *BTK* | *CRKL* | *FAM123B* | *GATA1* | *KDM6A* | *MYC* | *PAX5* | *RB1* | *TBX3* |
| *APC* | *C11ORF30* | *CRLF2* | *FAM46C* | *GATA2* | *KDR* | *MYCL1* | *PBRM1* | *REL* | *TET2* |
| *APCDD1* | *C17ORF39* | *CSF1R* | *FANCA* | *GATA3* | *KEAP1* | *MYCN* | *PDGFRA* | *RET* | *TGFBR2* |
| *AR* | *CARD11* | *CTCF* | *FANCC* | *GNA11* | *KIT* | *MYD88* | *PDGFRB* | *RICTOR* | *TIPARP* |
| *ARAF* | *CASP8* | *CTNNA1* | *FANCD2* | *GNA13* | *KLHL6* | *MYST3* | *PDK1* | *RNF43* | *TMPRSS2* |
| *ARFRP1* | *CBFB* | *CTNNB1* | *FANCE* | *GNAQ* | *KRAS* | *NBN* | *PIK3C2G* | *RPA1* | *TNFAIP3* |
| *ARID1A* | *CBL* | *CUL4A* | *FANCF* | *GNAS* | *LMO1* | *NCOR1* | *PIK3C3* | *RPTOR* | *TNFRSF14* |
| *ARID2* | *CCND1* | *CUL4B* | *FANCG* | *GPR124* | *LRP1B* | *NF1* | *PIK3CA* | *ROS1* | *TOP1* |
| *ASXL1* | *CCND2* | *CYP17A1* | *FANCI* | *GRIN2A* | *MAP2K1* | *NF2* | *PIK3CG* | *RUNX1* | *TP53* |
| *ATM* | *CCND3* | *DAXX* | *FANCL* | *GSK3B* | *MAP2K2* | *NFE2L2* | *PIK3R1* | *RUNX1T1* | *TRRAP* |
| *ATR* | *CCNE1* | *DDR2* | *FANCM* | *HGF* | *MAP2K4* | *NFKBIA* | *PIK3R2* | *SETD2* | *TSC1* |
| *ATRX* | *CD79A* | *DIS3* | *FAT3* | *HLA-A* | *MAP3K1* | *NKX2-1* | *PMS2* | *SF3B1* | *TSC2* |
| *AURKA* | *CD79B* | *DNMT3A* | *FBXW7* | *HRAS* | *MAP3K13* | *NOTCH1* | *PNRC1* | *SH2B3* | *TSHR* |
| *AURKB* | *CDC73* | *DOT1L* | *FGF10* | *IDH1* | *MCL1* | *NOTCH2* | *PPP2R1A* | *SMAD2* | *VHL* |
| *AXL* | *CDH1* | *EGFR* | *FGF12* | *IDH2* | *MDM2* | *NOTCH3* | *PRDM1* | *SMAD4* | *WISP3* |
| *BACH1* | *CDK12* | *EP300* | *FGF14* | *IGF1* | *MDM4* | *NOTCH4* | *PRKAR1A* | *SMARCA4* | *WT1* |
| *BAP1* | *CDK4* | *EPHA3* | *FGF19* | *IGF1R* | *MED12* | *NPM1* | *PRKDC* | *SMARCB1* | *XPO1* |
| *BARD1* | *CDK6* | *EPHA5* | *FGF23* | *IGF2* | *MEF2B* | *NRAS* | *PRSS8* | *SMARCD1* | *XRCC3* |
| *BCL2* | *CDK8* | *EPHB1* | *FGF3* | *IKBKE* | *MEN1* | *NSD1* | *PTCH1* | *SMO* | *ZNF217* |
| *BCL2L2* | *CDKN1B* | *ERBB2* | *FGF4* | *IKZF1* | *MET* | *NTRK1* | *PTEN* | *SOCS1* | *ZNF703* |
| *BCL6* | *CDKN2A* | *ERBB3* | *FGF6* | *IL7R* | *MITF* | *NTRK2* | *PTPN11* | *SOX10* |  |
| *BCOR* | *CDKN2B* | *ERBB4* | *FGF7* | *INHBA* | *MLH1* | *NTRK3* | *RAD50* | *SOX2* |  |
| *BCORL1* | *CDKN2C* | *ERG* | *FGFR1* | *IRF4* | *MLL* | *NUP93* | *RAD51* | *SPEN* |  |
| *BCR* | *CEBPA* | *ESR1* | *FGFR2* | *IRS2* | *MLL2* | *PAK3* | *RAD51B* | *SPOP* |  |
| *BLM* | *CHEK1* | *ETV1* | *FGFR3* | *JAK1* | *MPL* | *PAK7* | *RAD51C* | *SRC* |  |
