# Supplementary material for: Next-generation sequencing in advanced Chinese melanoma reveals therapeutic targets and prognostic biomarkers for immunotherapy
Source: Sci Rep. 2022 Jun 10;12:9559. doi: 10.1038/s41598-022-13391-y (PMC9187737; doi:10.1038/s41598-022-13391-y)
Supplement: Supplementary file 2 — Supplementary Table S2. [file 41598_2022_13391_MOESM2_ESM.docx]

**Next-generation Sequencing in advanced Chinese melanoma reveals therapeutic targets and prognostic biomarkers for immunotherapy**

Fuxue Huang^#1,2^, Jingjing Li^#1^, Xizhi Wen^#1^, Baoyan Zhu^1,3^, Wei Liu^1^, Jiuhong Wang^1^, Hang Jiang^1^, Ya Ding^*1^, Dandan Li^*1^, Xiaoshi Zhang^*1^

**Table S2** **Clinicopathological information of patients with immunotherapy**

| **Clinical information** | **N=25** |
| --- | --- |
| Age (range) | 55(29-87) |
| Sex (%) |  |
| Male | 17（68.0%） |
| Female | 8（32.0%） |
| Best response (%) |  |
| CR | 1（4.0%） |
| PR | 1（4.0%） |
| SD | 11（44.0%） |
| PD | 9（36.0%） |
| Subtype (%) |  |
| CSD/NCSD | 14（56.0%） |
| Mucosal | 8（32.0%） |
| Acral | 3（12.0%） |
